# HER-2 status of circulating tumor cells in a metastatic breast cancer cohort: A comparative study on characterization techniques

**DOI:** 10.1371/journal.pone.0220906

**Published:** 2019-09-04

**Authors:** Anja Brouwer, Bram De Laere, Pieter-Jan van Dam, Dieter Peeters, Jasper Van Haver, Ellen Sluydts, Ali El Moussaoui, Pauline Mendelaar, Jaco Kraan, Marc Peeters, Steven Van Laere, Luc Dirix

**Affiliations:** 1 Centre for Oncological Research (CORE), University of Antwerp, Antwerp, Belgium; 2 Department of Oncology, Antwerp University Hospital, Antwerp, Belgium; 3 Department of Medical Epidemiology and Biostatistics, Karolinska Institutet, Stockholm, Sweden; 4 HistoGeneX NV, Wilrijk, Antwerp, Belgium; 5 Department of Medical Oncology, Erasmus Medical Center, Rotterdam, The Netherlands; 6 Department of Oncology, GZA Hospitals Sint-Augustinus, Antwerp, Belgium; The Ohio State University, UNITED STATES

## Abstract

**Background:**

Personalized targeted treatment in metastatic breast cancer relies on accurate assessment of molecular aberrations, e.g. overexpression of Human Epidermal growth factor Receptor 2 (HER-2). Molecular interrogation of circulating tumor cells (CTCs) can provide an attractive alternative for real-time biomarker assessment. However, implementation of CellSearch-based HER-2 analysis has been limited. Immunofluorescent (IF) image interpretation is crucial, as different HER-2 categories have been described. Major questions in CTC research are how these IF categories reflect gene expression and amplification, and if we should consider ‘medium’ HER-2 expressing CTCs for patient selection.

**Methods:**

Tumor cells from spiked cell lines (n = 8) and CTCs (n = 116 samples) of 85 metastatic breast cancer patients were enriched using CellSearch. Comparative analysis of HER-2 expression by IF imaging (ACCEPT, DEPArray, and visual scoring) with qRT-PCR and *HER-2/neu* FISH was performed.

**Results:**

Automated IF HER-2-profiling by DEPArray and ACCEPT delivered comparable results. There was a 98% agreement between 17 trained observers (visual scoring) and ACCEPT considering HER-2^neg^ and HER-2^high^ expressing CTCs. However, 89% of HER-2^med^ expressing CTCs by ACCEPT were scored negative by observers. HER-2^high^ expressing tumor cells demonstrated *HER-2/neu* gene amplification, whereas HER-2^neg^ and HER-2^med^ expressing tumor cells and CTCs by ACCEPT were copy-number neutral. All patients with HER-2-positive archival tumors had ≥1 HER-2^high^ expressing CTCs, while 80% of HER-2-negative patients did not. High relative gene expression of HER-2 measured on enriched CTC lysates correlated with having ≥1 HER-2^high^ expressing CTCs.

**Conclusion:**

Automated images analysis has enormous potential for clinical implementation. HER-2 characterization and clinical trial design should be focused on HER-2^high^ expressing CTCs.

## Introduction

Breast cancer is a heterogeneous disease, with distinct subgroups based on histological type, grade, and hormone receptor status. Human epidermal growth factor receptor 2 (HER-2) overexpression accounts for 10–15% of the primary invasive breast cancers and is associated with a more aggressive phenotype and inferior prognosis. In patients with advanced disease, clinically relevant discrepancies can arise in HER-2 expression status compared to the localized setting [1–3]. Furthermore, patients often develop multiple lesions that might be composed of various tumor subclones harboring different molecular characteristics [4]. As the HER-2 status can be subjective to temporal heterogeneity, in part influenced by prior therapies, it stands to reason that repeated analysis is a prerequisite for precision medicine. However, the acquirement of metastatic tissue is not always feasible and not without risk for the patient [5].

Circulating Tumor Cells (CTCs), isolated from the blood of patients with metastatic cancer, hold considerable promise to provide a convenient and safe alternative for real-time and repeated tumor profiling. Before molecular characterization of CTCs can be used to discover predictive biomarkers, e.g. HER-2 receptor status, in-depth testing of analysis methods is essential. The CellSearch system is an FDA-cleared and widespread implemented platform for enumeration of CTCs, and HER-2-positive CTCs have been detected using HER-2 immunofluorescence (IF) phenotyping. Visual scoring of HER-2 on CellSearch IF images by individual observers has been performed in several studies [6–8]. Image interpretation is crucial, especially when using CTCs in interventional trials testing HER-2-directed therapies. Although trained observers can reach acceptable agreement using a predefined definition [9], visual scoring is not objective and independent image review is laborious. Recently, an objective analysis software for CellSearch IF images: Automated CTC Classification Enumeration and PhenoTyping (ACCEPT) has been made available, which is able to divide CTCs in HER-2^neg(ative)^, HER-2^med(ium)^, and HER-2^high^ expression [8]. One major question in CTC research is how these IF categories reflect gene expression and amplification. One study demonstrated that HER-2-positive CTCs based on visual scoring were HER-2 gene amplified [10]. Still the value of HER-2^med^ expressing CTCs has to be studied, as patients harboring these CTCs might as well benefit from HER-2-directed therapies. In this study we compare ACCEPT results with other IF imaging, quantitative reverse transcription (qRT)-PCR, and fluorescent *in-situ* hybridization (FISH).

## Methods

The ethical committee of the Antwerp University Hospital (UZA) and University of Antwerp (UA) approved this non-interventional study (UA A11-18). Images used in the observer study were obtained from samples of patients enrolled in studies of the Erasmus Medical Center Rotterdam (METC 2016–313 and METC 2009–405). Informed written consent was obtained from all patients. The raw data can be downloaded from S1 Dataset.

**Fig 1:**
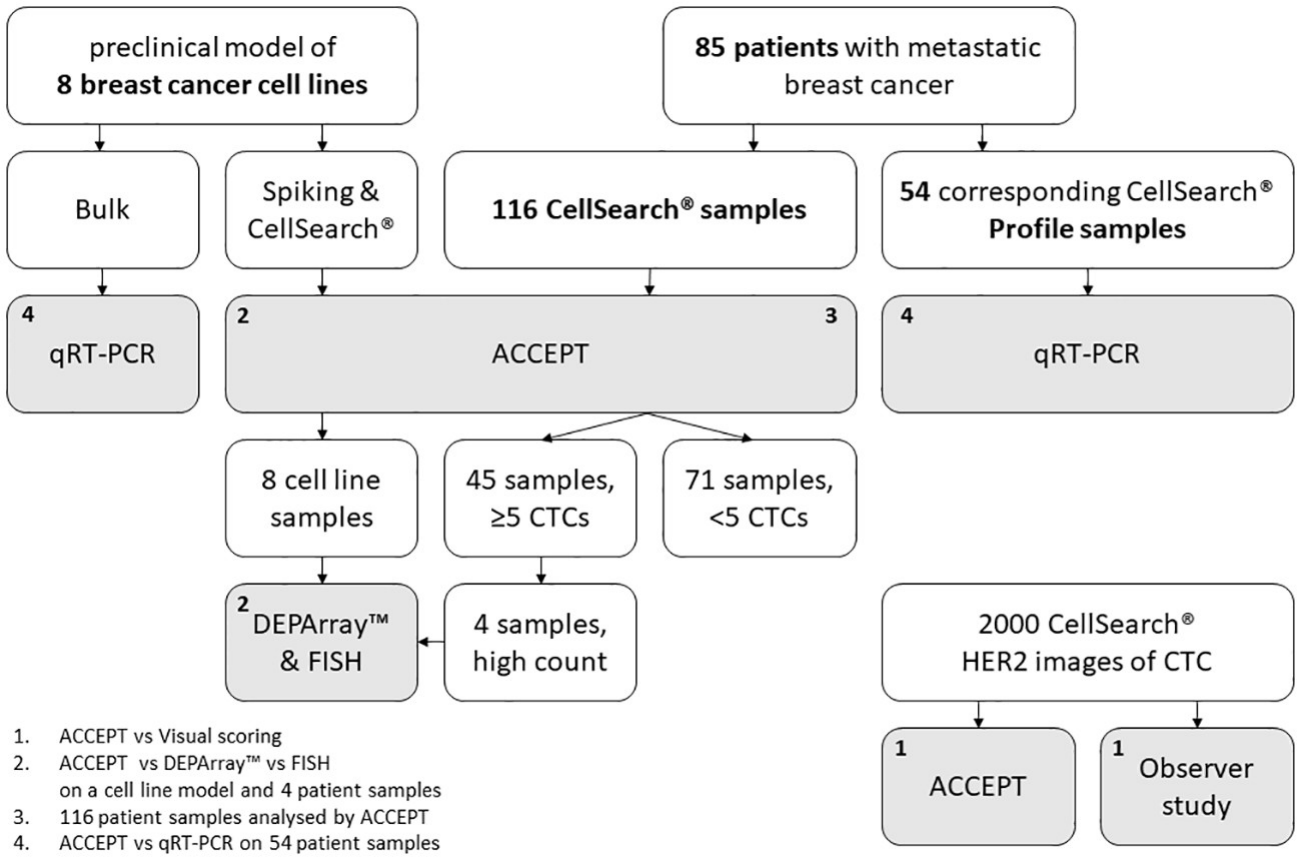
Schematic overview of samples and workflow.

A detailed description of all materials and methods is provided in S1 Supplementary Methods. A schematic overview of all samples and the workflow is depicted in Fig 1.

### Samples

The preclinical model utilizes eight breast cancer cell lines with increasing levels of HER-2 expression and/or amplification: MDA-MB-436, MCF-7, BT-20, MDA-MB-453, KPL-4, IBC-3, SUM190, and SKBR-3 [8, 9, 11–16]. Cultured tumor cells were spiked in 7.5mL Cell Save-collected healthy donor blood and subjected to the CellSearch CTC procedure (Menarini Silicon Biosystems Inc., Huntingdon Valley, PA, USA), with addition of the HER-2 phenotyping reagent (Menarini Silicon Biosystems Inc.). Briefly, the CellTracks Autoprep immunomagnetically enriches EpCAM-positive cells from blood and stains them with the nuclear dye DAPI, phycoerythrin conjugated antibodies against cytokeratin 8, 18 and 19 (CK-PE) and allophycocyanin conjugated antibody against the leukocyte specific marker CD45 (CD45-APC). The enriched cells are contained in a cartridge. Similarly, CTCs were enriched from 7.5 ml blood samples (n = 116) of 85 patients starting a new line of systemic therapy for metastatic breast cancer (MBC), who were recruited between 2012 and 2015 at the Oncology Center of GZA Hospitals Sint-Augustinus (Antwerp, Belgium), after written informed consent (Study UA A11-18)(S1 and S2 Tables). In total, 45/116 (38.8%) samples contained ≥5 CTC/7,5 ml blood. For the inter-observer concordance study, 17 international pathologist and scientists scored 2000 CellSearch HER-2-FITC thumbnail images of CTCs acquired from MBC patients who were enrolled, after written informed consent, in CTC studies at the Erasmus MC (Rotterdam, The Netherlands) (METC 2016–313 and METC 2009–405).

### IF imaging

Image-based HER-2 fluorescent intensities were analyzed using three methodologies (S3 Table). First, visual scoring, which classifies the cells into negative, 2+, and 3+ was employed, as previously described [9]. Using an online survey platform, CellTracks Analyzer II thumbnail images of CTCs (n = 2000) were reviewed by 17 international scientists and pathologists, who were trained to perform the visual HER-2 scoring. The obtained scores were benchmarked against the automated scoring results by ACCEPT [8]. ACCEPT was used to automatically analyze the raw TIFF images of every fluorescent filter (DAPI, PE, APC, and FITC) taken by the CellTracks Analyzer II (Menarini Silicon Biosystems Inc.) [8]. CTC identification and HER-2 IF intensity classification (HER-2^neg^, HER-2^med^, and HER-2^high^) was performed with gating and HER-2-FITC cut-off settings as previously described [8, 17]. Briefly, CTC gates are defined as: Mean Intensity CD45 ≤ 5, Mean Intensity DNA > 45, Mean Intensity CK > 60, 16 ≤ Size CK ≤ 400, DNA overlay CK > 0.2; and HER-2 cut-offs are: HER-2^neg^ (Mean Intensity HER-2 = 0), HER-2^med^ (<100), and HER-2^high^ (≥100). Thirdly, to validate objective IF scoring by ACCEPT, 7 CellSearch-enriched tumor cell lines and 4 CTC samples with high count were transferred to the DEPArray V2 system (Menarini Silicon Biosystems Inc.), as we have described previously [18]. Briefly, the loaded sample is automatically injected into the microchamber of a cartridge where single cells are trapped in one of 16,000 electrical cages. IF images of the entire surface area are taken and cells are automatically detected by the system, generating an image library and 40 parameters per individual cell. HER-2 scoring was performed using the relative fluorescent units (RFU) of the HER-2-FITC signal after background subtraction (i.e. Mean Intensity-bgsub parameter). A cut-off for HER-2 positivity was defined at >1185 RFUs. Using this cut-off, 95% of the analyzed cells within the theoretically expected HER-2-positive and -negative cell lines classified as positive and negative, respectively.

### FISH

A *HER-2/neu* FISH protocol was established using cell line models. CellSearch-enriched tumor cells (8 spiked cell lines) and patient CTCs (4 samples) were spinned on a Superfrost Plus slide (Fisherbrand) using a Slide carrier with a 1ml One-Funnel Cytochamber (cat. 1662 and 1663 resp., Hettich) and fixed in acetone at 4°C for 5 minutes. FISH on slide was performed using the DAKO IQFISH kit (Agilent), with adjusted protocol as described in S1 Supplementary Methods. Before and after FISH, slides were scanned on the BioView scan device with a specialized CTC protocol (BioView, Israel), in order to detect and map the tumor cells in the leukocyte background. HER-2 status was assessed according to the manufactures guidelines (Agilent).

### qRT-PCR

Besides HER-2 image analysis, CellSearch Profile-enriched tumor cell fractions (cell lines: n = 7, patient samples: n = 54) were subjected to HER-2 expression analysis, as described previously [19]. Samples were taken simultaneously with CellSearch CTC samples, to facilitate comparison between gene expression and IF. Briefly, 25% of the isolated RNA from the enriched fraction was subjected to complementary DNA (cDNA) synthesis and pre-amplification, using the RevertAid H Minus First Strand cDNA synthesis kit and TaqMan PreAmp master mix, respectively (Thermo Fisher Scientific #K1632 and #4488593). Pre-amplified cDNA was diluted 15x with 1xTE-buffer, after which qRT-PCR was performed for *ERBB2* as target gene, 3 housekeeping genes (*SDHA*, *HMBS* and *HPRT1*) to control for sample loading and RNA integrity, epithelial (*EPCAM*, *KRT19*) and leukocyte (*PTPRC*) markers to control for presence of epithelial and leukocyte content. *ERBB2* Cq value of every sample was normalized to the epithelial signal within that sample (dCq). All samples were further normalized to the calibrator (ddCq).

### Statistics

Correlations between the HER-2 analysis methods ACCEPT, DEPArray, and qRT-PCR was calculated using Pearson’s correlation coefficient. Fisher’s exact test was used to compare ACCEPT and qRT-PCR results within HER-2-negative and HER-2-positive patient groups.

## Results

### HER-2 protein expression and gene amplification

**Fig 2:**
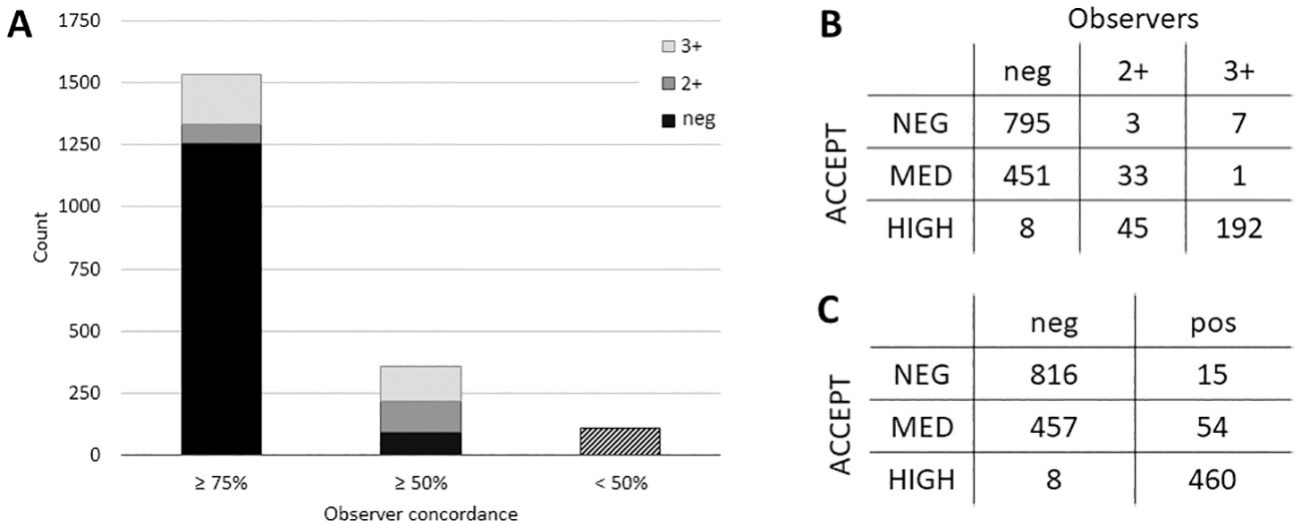
HER-2 scoring of 2000 CTC thumbnail IF images by 17 trained observers (0, 2+, 3+) versus ACCEPT (HER-2^neg^, HER-2^med^, HER-2^high^). **A**. ≥75% concordance between observers was reached for 1535 CTCs. For 358 CTCs agreement was reached between >50% of observers, and for 107 CTCs (dashed box) no agreement was reached **B**. HER-2 scores of 1535 CTCs given by ≥75% of observers versus ACCEPT. HER-2-negative CTCs according to observers were mainly scored HER-2^neg^ or HER-2^med^ by ACCEPT. HER-2^high^ expressing CTCs by ACCEPT were predominantly scored 2+ or 3+ by the observers. **C**. HER-2 scores of 1810 CTCs given by ≥75% of observers versus ACCEPT. HER-2^high^ expressing CTCs by ACCEPT were predominantly scored positive by the observers.

HER-2 IF image interpretation is crucial, especially when using CTCs in interventional trials testing HER-2-directed therapies. In our international observer study, 17 trained readers performed visual HER-2 scoring (negative, or positive: 2+ or 3+) of 2000 patient-derived thumbnail CellSearch HER-2 images, which was compared to the new objective ACCEPT algorithm (HER-2^neg^, HER-2^med^, and HER-2^high^). For 1535 CTCs (77%) a high concordance (i.e. >75%) between observers was reached, which was predominantly driven by agreement on HER-2 negativity (Fig 2A). According to ACCEPT, 860 (43.8%) CTCs were HER-2^neg^, 608 (31.0%) were HER-2^med^, and 495 (25.2%) were HER-2^high^ expressing CTCs. 37 CTCs were not detected by ACCEPT. When comparing the 1535 highly concordant scored CTCs with ACCEPT results, observers tend to score HER-2 expression on these CTCs lower than ACCEPT does (Fig 2B). Especially HER-2^med^ expressing CTCs were frequently scored as negative cells by observers. When merging the 2+ and 3+ scored CTCs into one HER-2-positive group, high concordance (i.e. >75%) was reached for 1843/2000 (92%) CTCs. 460/468 (98%) HER-2^high^ expressing CTCs were scored as positive by the observers, and 816/831 (98%) HER-2^neg^ CTCs were scored negative by the observers. Again, ACCEPT HER-2^med^ CTCs were scored negative in 457/511 (89%) cases (Fig 2C). These dim expressing CTCs are covered in the negative category of the visual scoring system, as it includes 0 and 1+ scores. Furthermore, there was a significant difference between the mean IF intensity of the HER-2^med^ CTCs scored negative (47.9 +/- 15.5) versus positive (82.1 +/- 13.1) on visual scoring. Inter-observer variability (Kappa test) was 0.636 for visual scoring in negative, 2+, and 3+ groups, and 0.785 for scoring in negative versus positive groups, which are both considered as ‘good’ according to the Koch and Landis classification [20]. Overall, we found high agreement between observers and ACCEPT regarding HER-2^neg^ and HER-2^high^ expressing CTCs, however ACCEPT HER-2^med^ CTCs appear negative on visual scoring. To further investigate this, we performed IF image analysis and FISH on a cell line model.

**Fig 3:**
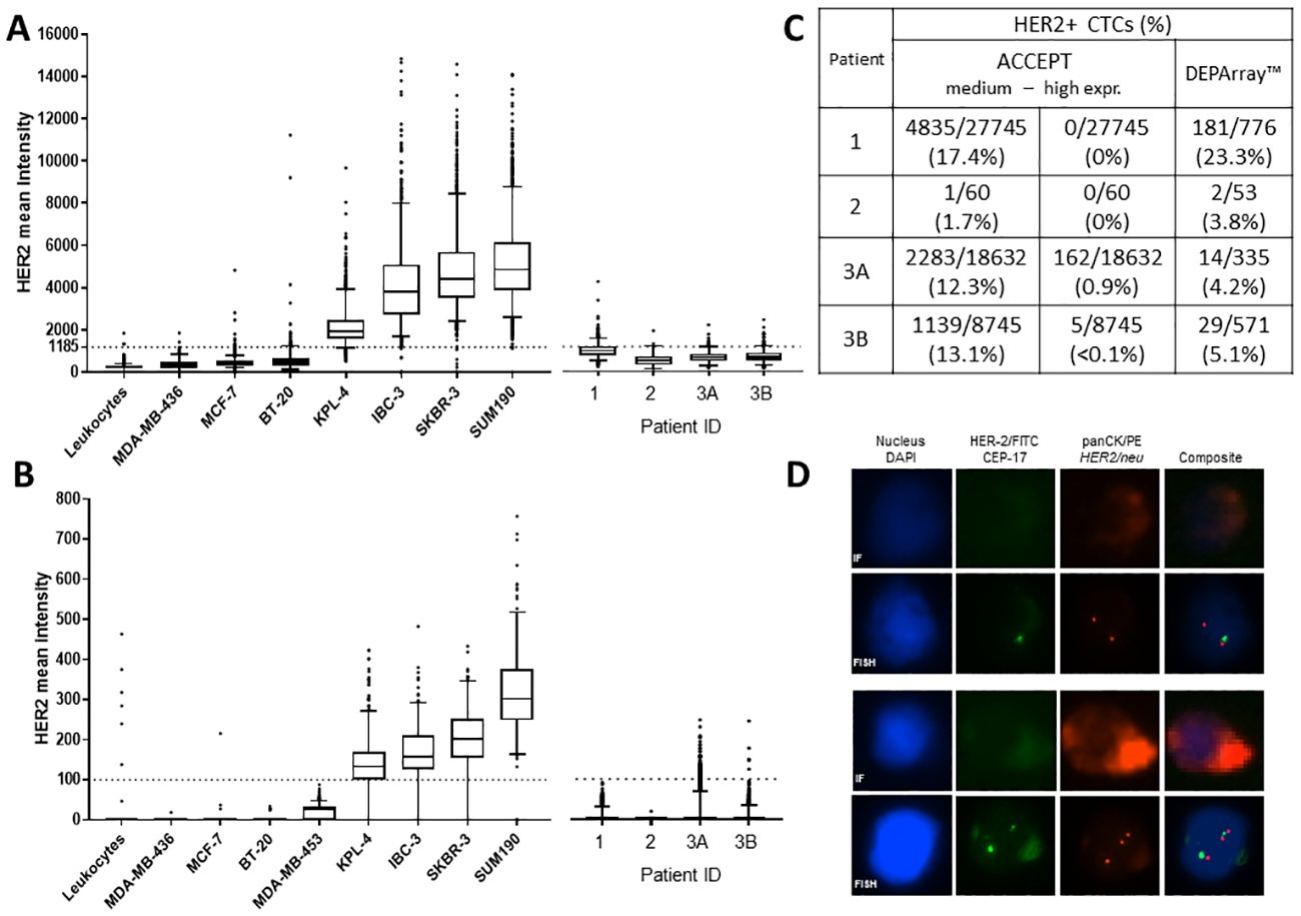
HER-2 IF and FISH on a cell line model and 4 patient samples. **A**. HER-2 mean intensity background subtracted (bgsub) for donor leukocytes, 3 HER-2-negative cell lines (MDA-MB-436, MCR-7, BT-20) and 4 HER-2-posititve cell lines (KPL-4, IBC-3, SKBR-3, SUM190), and 4 patient samples measured by DEPArray. The cut-off between HER-2-negative and -positive cells was defined at 1185 RFU. **B**. HER-2 mean intensities for donor leukocytes, 8 cell lines, and 4 patient samples measured by ACCEPT. HER-2^neg^, HER-2^med^ and HER-2^high^ expressing cells are defined as mean intensity = 0, ≤100, and >100 respectively [8]. **C**. Table showing the number and percentage of HER-2-positive CTCs in 4 patient samples based on ACCEPT and DEPArray. HER-2-positive cells are subdivided in HER-2^med^ and HER-2^high^ expressing cells using ACCEPT. **D**. BioView IF and FISH images of DEPArray-sorted HER-2-positive and -negative CTCs from patient 1. HER-2-negative CTCs are copy-number neutral (example shown). 7/24 (29%) HER-2-positive CTCs revealed a HER-2/CEP17 ration of 3:2, still being FISH negative (example shown).

Two automated image analysis methods, i.e. DEPArray and ACCEPT, were used to measure HER-2 IF signal on CellSearch-enriched samples of eight breast cancer cell lines with incremental HER-2-FITC intensity, categorized as HER-2^neg^ (MCF-7, MDA-MB-436, and BT-20), HER-2^med^ (MDA-MB-453), and HER-2^high^ (KPL-4, SUM190, IBC-3, and SKBR-3) according to literature [8, 9, 13] (Fig 3A and 3B). ACCEPT analysis of CellSearch Analyzer II raw images demonstrated absence of HER-2-FITC signal in 8409/8434 (99.7%) leukocytes and 496/509 (97.4%) negative cell line cells (Fig 3B). MDA-MB-453 cells were HER-2^med^ expressing in 368/640 (57.5%), the rest being HER-2^neg^. Within the HER-positive cell lines KPL-4, IBC-3, SKBR-3, and SUM190 we observed an incremental increase in the median HER-2-FITC intensity, with 415/542 (76.6%), 187/212 (88.2%), 189/208 (90.9%) and 220/228 (96.5%) cells, respectively, being classified as HER-2^high^ expressing cells (Fig 3B).

Overall, when comparing both DEPArray and ACCEPT cell line data, a comparable gradient in the mean HER-2 expression levels was observed (Pearson *r* = 0.96, *p* = 0.0001). As the DEPArray system is able to recover individual CTCs, the found cut-off can be used to sort samples into different HER-2 categories for downstream analysis.

To infer whether increased HER-2-FITC IF signals find their origin in gene amplification, we applied *HER-2/neu* FISH on the CellSearch-enriched fractions. In all samples, analyzed leukocytes (n = 20) demonstrated a copy-number neutral *HER-2/neu* status (S1 Fig). A similar observation was made in HER-2-negative cell lines MDA-MB-436, MCF-7, and BT-20, (n = 10 per cell line). High gene amplification was detected in all visualized cells of HER-2-positive cell lines KPL-4, IBC-3, SKBR-3, and SUM190 (n = 7 for KPL-4, n = 10 for IBC3, SKBR-3, and SUM190), with mean HER-2/CEP17 ratios of 5.5 (KPL-4), 6.3 (IBC-3), 8.3 (SKBR-3), and 4.5 (SUM190). Medium cell line MDA-MB-453 had on average 6 HER-2 and 3 CEP17 copies, being scored as borderline (S1 Fig).

Cell line results were confirmed in an explorative study on four patient samples with high CTC count. The majority of CTCs were HER-2-negative using DEPArray and ACCEPT (Fig 3C). With DEPArray image analysis, 23.3% of CTCs in patient sample 1 exceeded the HER-2-positivity threshold. A comparable HER-2^med^ expressing CTC rate was detected by ACCEPT. In patient samples 3 and 4 we observed 2283 (12.3%) and 1139 (13.1%) HER-2^med^ expressing CTCs, respectively. In all CellSearch patient samples no HER-2 amplification was observed. Additionally, CTCs from patient 1 just exceeding the positivity threshold were DEPArray-sorted. *HER-2/neu* FISH analysis showed no amplification in these CTCs, although 7/24 (29%) cells visualized, revealed a HER-2/CEP17 ratio of 3:2 (i.e. non-amplified according to HER-2-FISH guidelines) (Fig 3D).

Our finding that HER-2^med^ expressing cells are mainly scored as negative using visual scoring, and are not HER-2-amplified, is in line with results from a large patient cohort where only positive CTCs by visual scoring were HER-2-amplified [10].

### HER-2 analysis in a MBC patient cohort

**Fig 4:**
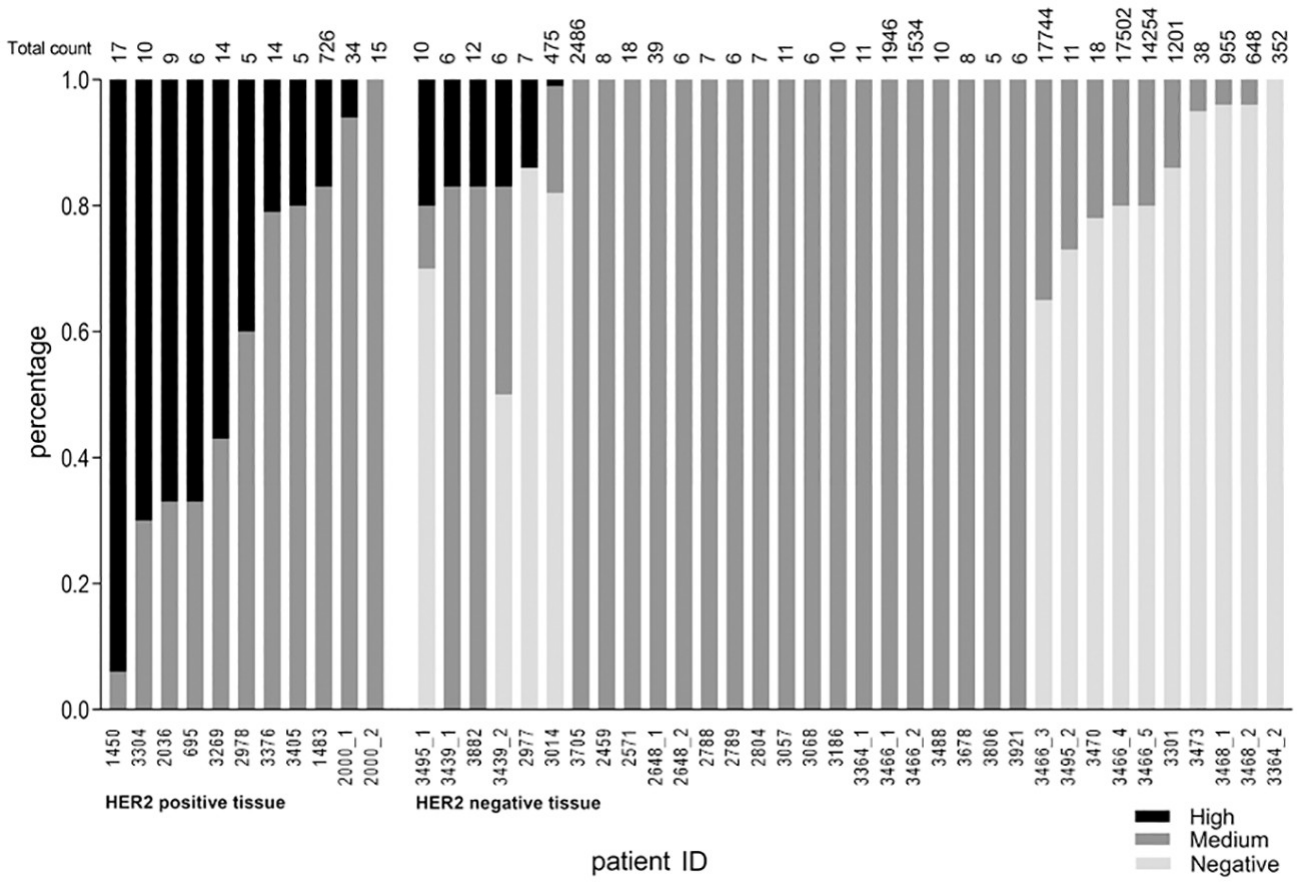
HER-2 IF scoring by ACCEPT of a MBC patient cohort. Percentage of HER-2^neg^, HER-2^med^, and HER-2^high^ expressing CTCs in a MBC cohort divided in patients with HER-2-positive or -negative tissue (primary tumor and/or metastasis) samples. Total CTC count per sample is depicted on top. HER-2^high^ expressing CTCs are present in at least 1 sample of 10/10 (100%) HER-2-positive patients, and 5/25 (20%) HER-2-negative patients.

Using the objective analysis software of ACCEPT, we studied the distribution of HER-2^neg^, HER-2^med^, and HER-2^high^ expressing CTCs in 45 CellSearch CTC samples containing ≥5 CTCs from 35 MBC patients. 11/45 (24%) samples came from 10 patients with a HER-2-positive primary tumor and/or metastasis, and 34/45 (76%) samples came from 25 patients with a HER-2-negative primary tumor and/or metastasis (Fig 4, S1 Table). In the HER-2-negative patient group, all first blood samples were taken at the start of a new line (1^st^-3^rd^) of therapy for MBC. None of these patients received any anti-HER-2 directed therapy. From HER-2-postitive patients, all samples (except from patient 2000) were taken at first line of therapy for MBC and none of them were at that moment treated with anti-HER-2 directed therapy, due to various reasons (i.e. *de novo* MBC; adjuvant trastuzumab had already stopped; or in 1 patient no anti-HER-2 directed therapy had been added to the adjuvant treatment). Sample 2000_1 was taken at the start of the second line of therapy, however the first line did not include trastuzumab. During further treatment this patient did receive anti-HER-2 directed therapy, as was the case when sample 2000_2 was taken (S2 Table). We observed in both groups heterogeneous HER-2 expression patterns. All 10 (100%) patients with HER-2-positive MBC, had ≥1 HER-2^high^ expressing CTCs, while in the HER-2-negative MBC patients, this was in 5/25 (20%) patients (Fisher exact: *p < 0*.*0001*). Overall, 37% of patients harbored >10% HER-2^high^ expressing CTCs. However, when combining HER-2^med^ and HER-2^high^ expressing CTCs, this was 94% of patients. This is comparable with recent data of 132 patients (39% HER-2-positive and 61% HER-2-negative patients), where 89.4% of patients had HER-2^med^ and/or HER-2^high^ expressing CTCs [8].

Focusing on patients with multiple sampling, comparable HER-2^neg^, HER-2^med^, and HER-2^high^ CTC counts were observed. However, in patient 2000, diagnosed with HER-2-positive *de novo* MBC, HER-2^high^ expressing CTCs were eliminated after anti-HER-2 directed therapy. Similarly, in patient 3495, who was diagnosed with HER-2-negative (IHC 2+/FISH-negative) *de novo* MBC, 20% of the CTCs were HER-2^high^ expressing at the start of an aromatase inhibitor. Although the CTC burden was comparable, HER-2^high^ expressing CTCs disappeared completely after 6 weeks on therapy (Fig 4, S2 Table).

Surprisingly, in all 71 samples containing <5 CTCs, we found no HER-2^neg^ CTCs and solely HER-2^med^ and HER-2^high^ expressing CTCs, independent of the primary tumor or metastasis status (S2 Fig). A similar observation was made by Zeune *et al*, with samples containing solely HER-2^med^ and/or HER-2^high^ CTCs all having ≤6 CTCs in total [8]. When analyzing leukocytes, we found in all samples up to 5% of leukocytes did express some HER-2. This physiological phenomenon will not affect IF image-based HER-2 analysis of CTCs, as CTCs and leukocytes are measured individually [21].

**Fig 5:**
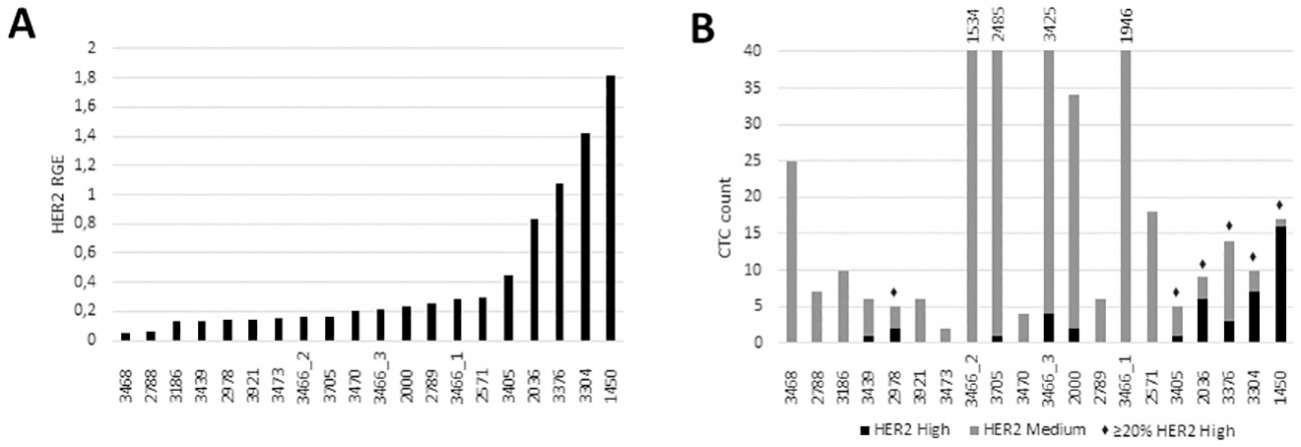
HER-2 protein (ACCEPT) versus mRNA (qRT-PCR) expression of MBC patient samples. **A**. Increasing *ERBB2* (HER-2) relative gene expression (RGE) corrected for CTC content of 20 patient samples with ≥5 CTCs. **B**. Corresponding HER-2^med^ and HER-2^high^ expressing CTC count by ACCEPT of 20 patient samples with ≥5 CTCs. For four samples the total number of HER-2^med^ expressing cells is given on top. For six samples ≥20% of total CTC count were HER-2^high^ expressing CTCs (◆).

We aimed to validate the ACCEPT results by applying gene expression analysis on enriched CTC fractions of blood samples taken simultaneously. ACCEPT image analysis and qRT-PCR data on CellSearch Profile-enriched CTC fractions were available for 7 cell lines and 54 patient samples. *ERBB2* (*HER-2/neu*) relative gene expression (RGE) levels in cell lines (S3 Fig) correlated with mean intensity HER-2 IF data, as measured by DEPArray (*r = 0*.*97*, *p = 0*.*0002*) and ACCEPT (*r = 0*.*96*, *p = 0*.*0007*) image analysis. *ERBB2* expression, corrected for CTC content, in 20 patient samples with ≥5 CTCs (range 5–17502) demonstrated variable *ERBB2* RGE (Fig 5), but correlated well with ACCEPT data (*r* = 0.8255, *p* < 0.00001). Focusing on HER-2^high^ expressing CTCs, comparative analysis with *ERBB2* RGE data demonstrated how 3/10 (30%) samples with low *ERBB2* RGE (<0.2, i.e. below median RGE) contained HER-2^high^ expressing CTCs (range 1–2), whereas HER-2^high^ expressing CTCs (range 1–16) were present in 7/10 (70%) samples with high *ERBB2* RGE (>0.2) (Fisher exact: *p = 0*.*1789*). When focusing on the samples with a highest *ERBB2* expression (RGE >0.34, i.e. above the third quartile) 5/5 patients harbored >20% (i.e. above the third quartile) HER-2^high^ expressing CTCs, while in the other group this was in 1/15 of patients (Fisher exact: *p = 0*.*0004*). Moreover, 5/5 patients with the highest *ERBB2* expression had ≥1 HER-2^high^ expressing CTC, while this was 5/15 in the group with a lower *ERBB2* RGE (Fisher exact: *p = 0*.*016*). We did not observe a correlation between ACCEPT and qRT-PCR data in samples with <5 CTCs. Taken together, this demonstrates that in patients with ≥5 CTCs, qRT-PCR on CellSearch-enriched samples can identify samples containing HER-2^high^ expressing CTCs.

## Discussion

Personalized targeted treatment of patients with MBC relies on the accurate assessment of specific molecular aberrations in tumor cells, e.g. the overexpression of the transmembrane HER-2 receptor. To circumvent potential clinically-relevant discordances in HER-2 receptor status between archival primary tumor tissue and metastatic lesions, the molecular interrogation of FDA-cleared CellSearch-enriched CTCs can provide an attractive alternative for real-time biomarker assessment [1–3]. However, implementation of CellSearch-based HER-2 analysis using visual scoring has been limited. Most recently an objective analysis software has been made available [8], which we compared to other CTC analysis techniques.

Our observer study demonstrated high agreement between the observers and ACCEPT considering the HER-2^neg^ and HER-2^high^ expressing CTCs, while HER-2^med^ expressing CTCs by ACCEPT were scored negative by the observers in 89% of CTCs. Moreover, we show that HER-2^med^ expressing cell line cells and patient CTCs, did not show HER-2/*neu* gene amplification, which is in agreement with literature, were MDA-MB-453 was scored IHC and FISH negative [8, 9, 11–13, 22]. Both results are in line with data from a large patient cohort where negative CTCs by visual scoring were HER-2 copy number neutral [10]. As patients only receive HER-2-directed therapy when HER-2 overexpression is proven on tissue samples (i.e. IHC 3+ or FISH+), one might argue on the clinical benefit of treating patients harboring HER-2^med^ expressing CTCs.

When inferring the prevalence of HER-2^neg^, HER-2^med^, and HER-2^high^ expressing CTCs in our patient cohort, we found that one third harbored >10% HER-2^high^ expressing CTCs, while almost all patients harbored HER-2^med^ expressing CTCs. This is comparable with recent data on a similar cohort of 132 patients [8]. In daily clinic, HER-2 overexpression (IHC 3+ or FISH+) is only present in a minority of patients with primary invasive breast cancer, although a higher incidence of HER-2-positivity is seen in MBC (26,3% in stage IV versus 15% in stage I-III patients) [23]. This prevalence is in line with the percentage of patients with HER-2^high^ expressing CTCs in both our and the MBC cohort examined by Zeune *et al* [8]. Taken together, we suggest that HER-2^high^ expressing CTCs might be more clinically relevant than HER-2^med^ expressing CTCs.

Besides all HER-2-positive patients, also 5/25 HER-2-negative patients harbored ≥1 HER-2^high^ expressing CTCs based on ACCEPT. This suggests either a shift in HER-2 status in these 5 patients, or outgrowth of a minor HER-2-positive subclone not detected with FISH on tissue samples. The latter has been demonstrated with FISH on DEPArray sorted primary tumor samples [24]. One should realize that HER-2 expression on tissue samples is often heterogeneous and are given IHC scores of 1+ or 2+. In our cohort, 3 out of 5 patients with discrepant HER-2 status were assigned IHC 1+ or 2+, suggesting some HER-2-positive tumor cells were already present at baseline. In general, IHC status (i.e. more homogeneous 0 or 3+, or more heterogeneous 1+ or 2+) of the archival tumor was not related to the degree of heterogeneity we found in the CTC samples. Acquisition (i.e. clonal selection/expansion) of HER-2 gene amplification in CTCs has reported to be associated with cancer progression [25]. Still, 80% of patients with a HER-2-negative primary tumor did not harbor any HER-2^high^ expressing CTCs. We argue that in these patients a major clinical impact of HER-2-directed monotherapy cannot be expected.

Clinical trials incorporating quantitative HER-2 analysis on CTCs might learn us the clinical validity of both HER-2^med^ and HER-2^high^ expressing CTCs. The ongoing DETECT III trial aims to demonstrate the benefit from Lapatinib therapy in initially HER-2-negative patients, who are HER-2-positive on CTCs [7]. The CirCe T-DM1 trial showed that *HER-2/neu* gene amplification in CTCs from 7 HER-2-negative MBC patients occurs in a minor CTC subpopulation [26]. Overall a low response rate was reported (1/7), questioning the clinical utility of anti-HER-2 therapy in patients with HER-2 amplification in a minor subset. Another phase II trial tested effectiveness of Lapatinib in MBC patients with HER-2-negative primary tumors and HER-2-positive CTCs analyzed by visual scoring of CellSearch images and FISH [27]. 7/96 patients, harboring 2–5 CTCs, were eligible (i.e. ≥50% of CTCs were HER-2-IF positive, and 1 sample was FISH-positive). No objective tumor responses occurred in this population, underlining the importance of patient selection for such trials. Based on our findings this should be patients with ≥5 CTCs and at least one HER-2^high^ expressing CTC. To enhance clinical utility of CTC-based therapy selection, it is important to consider improved quality control, validation, and standardization for HER-2 characterization and scoring on CTCs, as is required for HER-2 diagnostics on tissue. Objective image analysis is key start.

Liquid biopsies have the major advantage that they can be taken easily and repeatedly. The ability to detect *ERBB2* gene amplifications in plasma has already been proven [28], however no trials testing anti-HER-2 directed therapy in MBC based on HER-2 alterations in cell free (cf)DNA have been performed. Although, efforts have been made in gastric cancer [29]. For both cfDNA and CTCs (independent of the enrichment technique, i.e. EpCAM based or marker free) applies: a standardized biomarker should be tested in the right patient population in a four-armed randomized trial [30, 31] to proof its utility in distinguishing between patients that will or will not benefit from specific therapies.

## Conclusions

Our data shows that HER-2 characterization on CTCs should be focused on HER-2^high^ expressing CTCs in patient samples containing ≥5 CTCs. Although CTC-derived HER-2 expression in patients is heterogeneous, the prevalence of patients with ≥1 HER-2^high^ expressing CTCs better reflects the incidence of HER-2-positive MBC seen in the clinic. Additionally, we have demonstrated that only HER-2^high^ expressing tumor cells harbor amplification of the *HER-2/neu* gene, and samples containing HER-2^high^ expressing CTCs show high relative gene expression of HER-2 on qRT-PCR. For both these downstream techniques, prior CTC enrichment is necessary, involving extra cost and labor. Therefore, straightforward automated images analysis has enormous potential for clinical implementation. When focusing on the right patient population, CTC-direct anti-HER-2 therapy might proof itself in clinical trials.

## Supporting information

S1 Table HER-2 tissue status. IHC (0–3+) and FISH (0 = negative, 1 = positive) results for primary tumor (PT) and metastatic tissue (MET) per patient.
(TIF)

S2 Table Line of treatment at time of CTC enumeration. In the HER-2-negative patient group, all first blood samples were taken at the start of a new line (1^st^-3^rd^) of therapy for MBC. None of these patients received any anti-HER-2 directed therapy. From the HER-2-postitive patients, all samples were taken at first line of therapy for MBC, but sample 2000_1 (start of the second line), and none of them were at that moment treated with anti-HER-2 directed treatment. Sample 2000_2 was taken at the start of the fifth line of therapy, after prior anti-HER-2 directed therapy.
(TIF)

S3 Table Methodologies and cut-offs used for image-based analysis of HER-2 fluorescent intensities. (TIF)

S1 Fig BioView IF and FISH images of leukocytes and cell line cells. IF composite image is taken before FISH. Secondly, Nucleus/DAPI, CEP-17/SpectrumGreen, *HER-2-neu*/SpectrumOrange, and the composite images are shown. Leukocytes, MDA-MB-436, MCF-7, and BT-20 cells demonstrated a copy-number neutral *HER-2/neu* status. Mean HER-2/CEP17 ratios for amplified cell lines were 5.5 (KPL-4), 6.3 (IBC-3), 8.3 (SKBR-3), and 4.5 (SUM190). Medium cell line MDA-MB-453 had on average 6 HER-2 and 3 CEP17 copies.
(TIF)

S2 Fig ACCEPT results in <5 CTC patient samples. HER-2^neg^, HER-2^med^, and HER-2^high^ expressing CTC count in a MBC cohort with samples <5CTC, divided in patients with HER-2-positive or -negative tissue (primary tumor and/or metastasis) samples.
(TIF)

S3 Fig HER-2 gene expression. *ERBB2* relative gene expression (RGE) corrected for housekeeping gene expression, of bulk samples from 7 cell lines.
(TIF)

S1 Supplementary Methods (PDF)

S1 Dataset RAWdata_HER2_CTC. (ZIP)
