# Supplementary figures and images for: HER-2 status of circulating tumor cells in a metastatic breast cancer cohort: A comparative study on characterization techniques
Source: PLoS One. 2019 Sep 4;14(9):e0220906. doi: 10.1371/journal.pone.0220906 (PMC6726188; doi:10.1371/journal.pone.0220906)

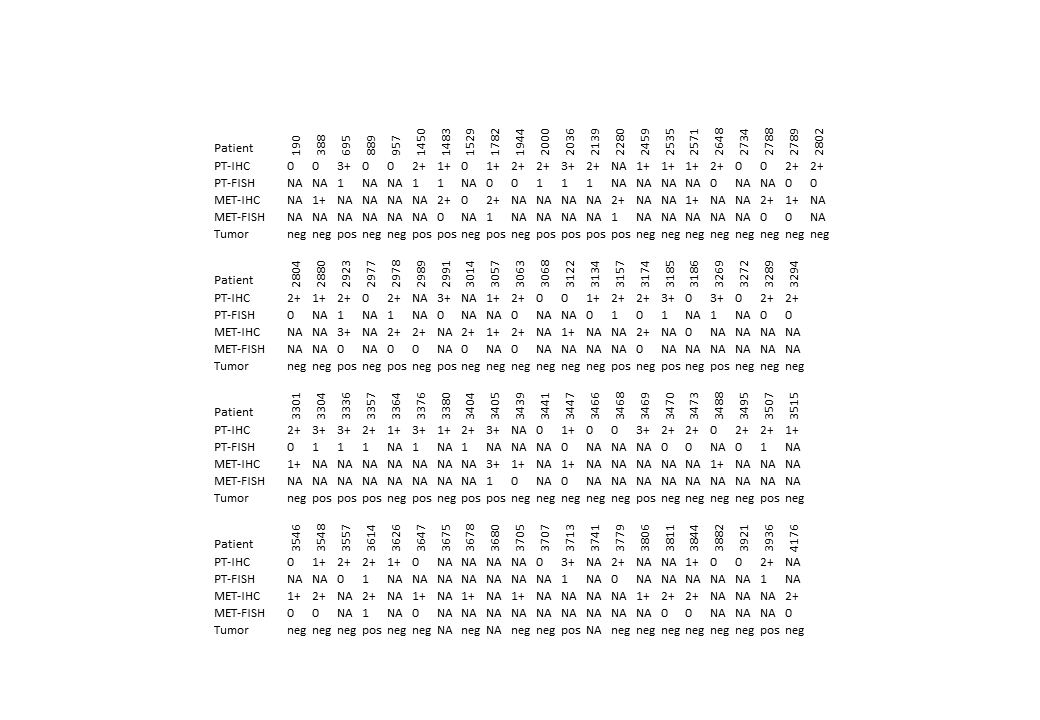

Supplement: S1 Table — IHC (0–3+) and FISH (0 = negative, 1 = positive) results for primary tumor (PT) and metastatic tissue (MET) per patient. (TIF) [file pone.0220906.s001.tif]

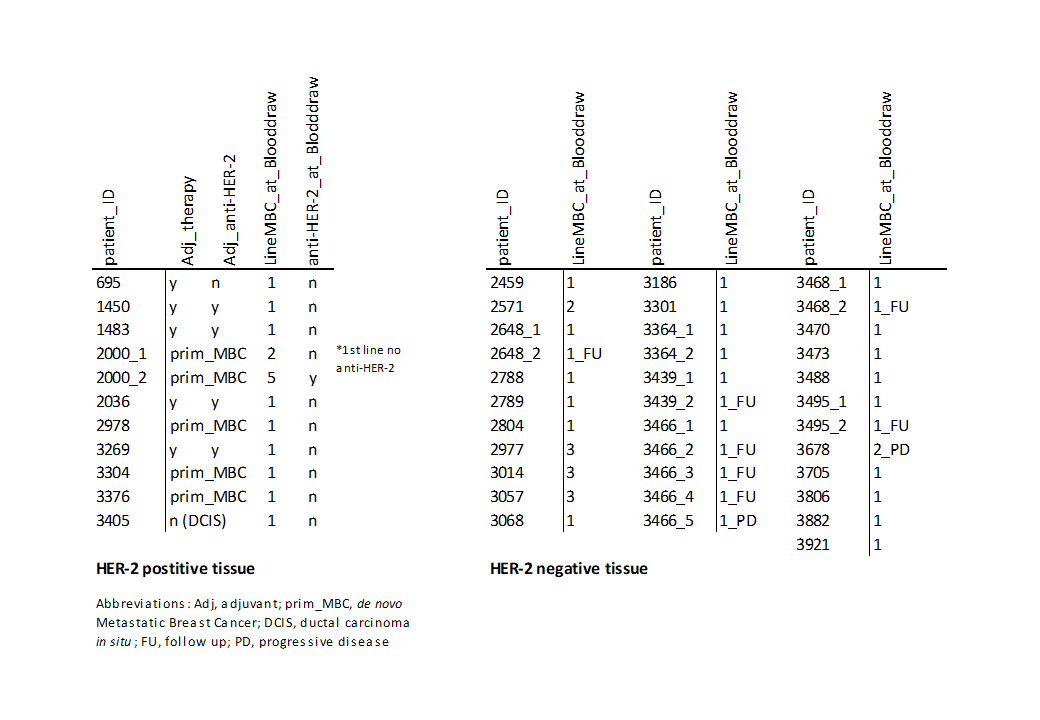

Supplement: S2 Table — In the HER-2-negative patient group, all first blood samples were taken at the start of a new line (1st-3rd) of therapy for MBC. None of these patients received any anti-HER-2 directed therapy. From the HER-2-postitive patients, all samples were taken at first line of therapy for MBC, but sample 2000_1 (start of the second line), and none of them were at that moment treated with anti-HER-2 directed treatment. Sample 2000_2 was taken at the start of the fifth line of therapy, after prior anti-HER-2 directed therapy. (TIF) [file pone.0220906.s002.tif]

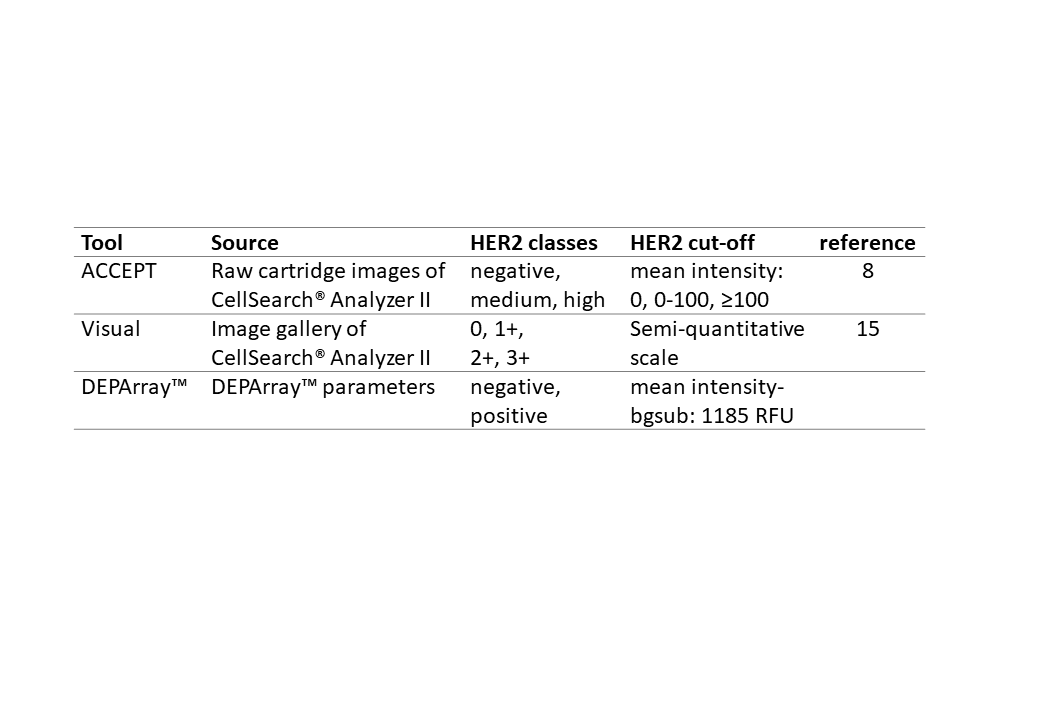

Supplement: S3 Table — (TIF) [file pone.0220906.s003.tif]

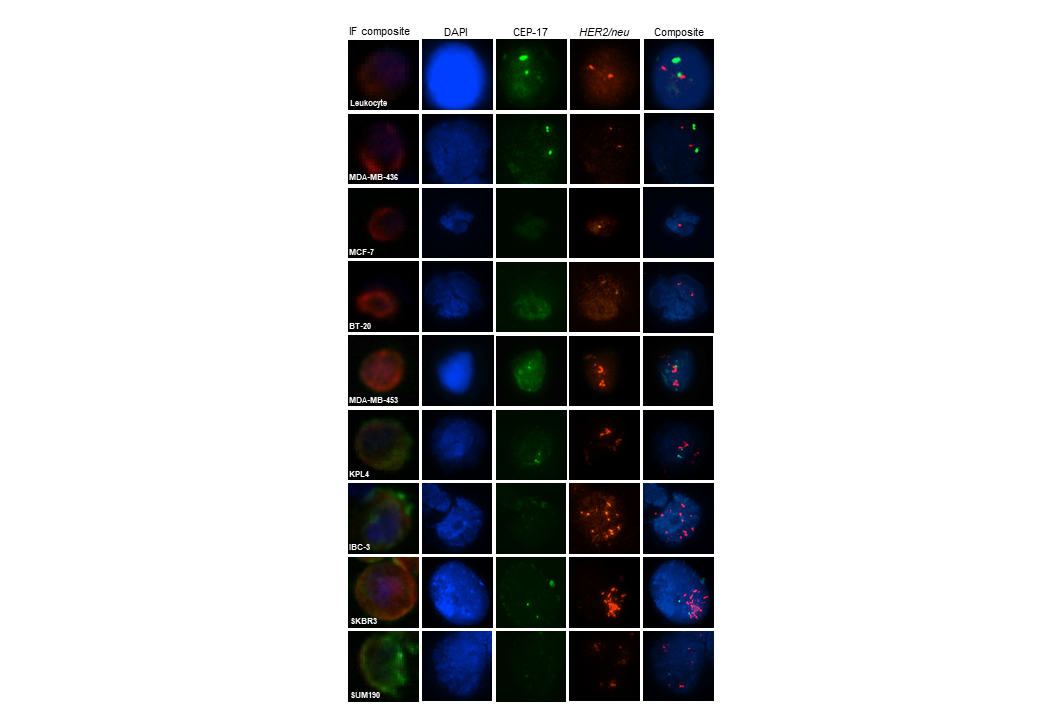

Supplement: S1 Fig — IF composite image is taken before FISH. Secondly, Nucleus/DAPI, CEP-17/SpectrumGreen, HER-2-neu/SpectrumOrange, and the composite images are shown. Leukocytes, MDA-MB-436, MCF-7, and BT-20 cells demonstrated a copy-number neutral HER-2/neu status. Mean HER-2/CEP17 ratios for amplified cell lines were 5.5 (KPL-4), 6.3 (IBC-3), 8.3 (SKBR-3), and 4.5 (SUM190). Medium cell line MDA-MB-453 had on average 6 HER-2 and 3 CEP17 copies. (TIF) [file pone.0220906.s004.tif]

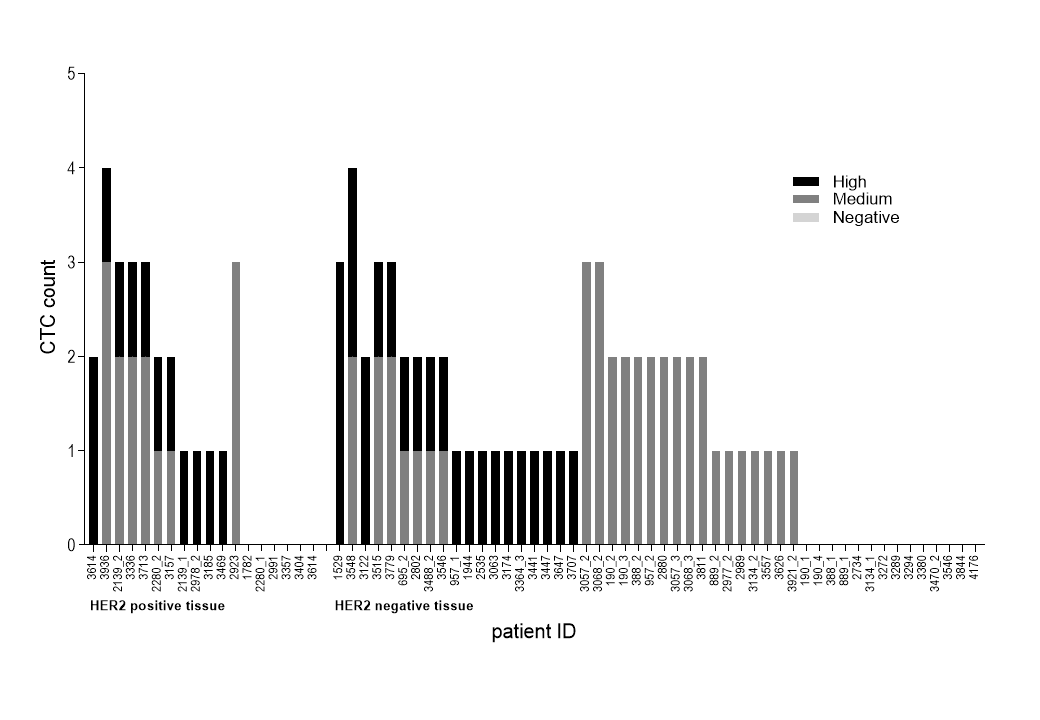

Supplement: S2 Fig — HER-2neg, HER-2med, and HER-2high expressing CTC count in a MBC cohort with samples <5CTC, divided in patients with HER-2-positive or -negative tissue (primary tumor and/or metastasis) samples. (TIF) [file pone.0220906.s005.tif]

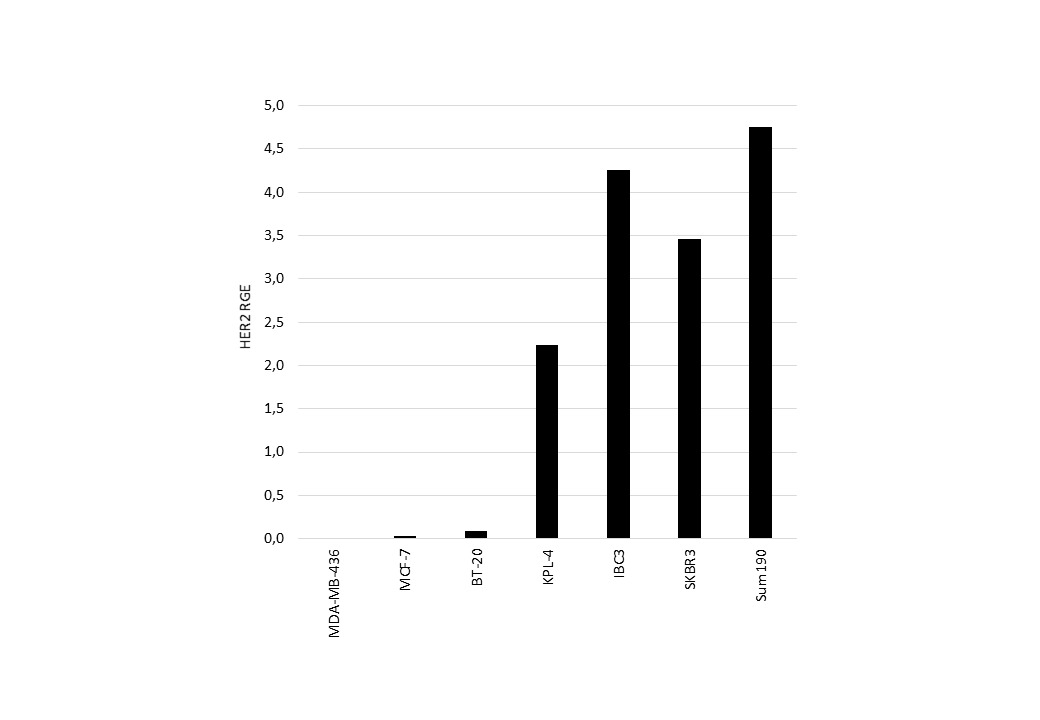

Supplement: S3 Fig — ERBB2 relative gene expression (RGE) corrected for housekeeping gene expression, of bulk samples from 7 cell lines. (TIF) [file pone.0220906.s006.tif]
