# Supplementary material for: HER-2 status of circulating tumor cells in a metastatic breast cancer cohort: A comparative study on characterization techniques
Source: PLoS One. 2019 Sep 4;14(9):e0220906. doi: 10.1371/journal.pone.0220906 (PMC6726188; doi:10.1371/journal.pone.0220906)
Supplement: S1 Supplementary Methods — (PDF) [file pone.0220906.s007.pdf]

Supplementary Methods

Samples

The preclinical model consists of eight breast cancer cell lines with increasing levels HER-2 expression and/or amplification: MDA-MB-436, MCF-7, BT-20, MDA-MB-453, KPL-4, IBC-3, SUM190, and SKBR-3 [8, 9, 11-16]. SUM190 and IBC-3 were cultured in Ham's F12 Nutrient Mixture (Life Technologies: 21765-029) with 1% penicillin/streptomycin/L-glutamine (Life Technologies: 10378-016), 1% anti/anti (Life Technologies: 15240-062), 5% FBS (Life Technologies: 10270-106), 1% HEPES buffer 1M (Life Technologies: 15630-056), 1µg/ml hydrocortisone (Sigma-Aldrich: H4001-1G), and 5µg/ml insulin solution (Sigma-Aldrich: I9278-5ML). MCF-7, MDA-MB-436, BT-20, and KPL-4 were cultured in RPMI (Life Technologies: 11835-063), and SKBR-3 in McCoy's (Life Technologies: 16600-082), all with 1% penicillin/streptomycin/L-glutamine, 1% anti/anti, and 10% FBS. Tumor cells were spiked in CellSave® tubes containing 7.5 ml donor blood and subjected to the CellSearch® system (Menarini Silicon Biosystems Inc., Huntingdon Valley, PA, USA), with addition of a FITC-bound HER-2 antibody (Menarini Silicon Biosystems Inc.). Similarly, CTCs were enriched from 7.5 ml blood samples (n=116) of 85 patients with metastatic breast cancer (MBC), who were recruited between 2012 and 2015 at the Oncology Center of GZA Hospitals Sint-Augustinus (Antwerp, Belgium), after written informed consent (Study UA A11-18)(Supplementary Table S2). In total, 45/116 (38.8%) samples contained ≥5 CTC/7,5 ml blood. For the inter-observer concordance study, 17 international pathologist and scientists scored 2000 CellSearch® HER-2-FITC thumbnail images of CTCs acquired from MBC patients who were enrolled, after written informed consent, in several CTC studies at the Erasmus MC (Rotterdam, The Netherlands). For this part we included all single, fully visible CTC images of selected patients (known to be CTC-HER-2 positive or negative) regardless of image quality.

HER-2 IF scoring methods

Image-based HER-2 fluorescent intensities were analyzed using three methodologies.

Tool	Source	HER-2 classes	HER-2 cut-off	reference
Visual	Image gallery of CellSearch® Analyzer II	0, 1+, 2+, 3+	Semi-quantitative scale	9
ACCEPT	Raw cartridge images of CellSearch® Analyzer II	negative, medium, high	mean intensity: 0, 0-100, ≥100	8
DEPArray™	DEPArray™ V2 parameters	negative, positive	mean intensity-bgsub: 1185 RFU	

First, a semi-quantitative scale, which classifies the cells into 0, 1+, 2+, and 3+ was employed, as previously described [9]. 0 and 1+ cells are considered negative. They have a pixelated cell staining with a grey and pixelated background. While 2+ and 3+ cells have sharp edge and fluent cell staining with a dark to completely black background respectively. Using an online survey platform, CellTracks Analyzer II® CTC images (n=2000) were reviewed by 17 international scientists and pathologists, who were trained to perform the visual semi-quantitative HER-2 scoring. The obtained scores were benchmarked against the automated scoring results by ACCEPT [8].

Secondly, ACCEPT was used to automatically analyze the raw TIFF images from the CellTracks Analyzer II® (Menarini Silicon Biosystems Inc.)[8]. CTC identification, and classification of HER-2 intensity levels (negative, medium, and high) was performed with gating and HER-2-FITC cutoff settings as previously described [8, 17]. It generates multiple parameters on shape and fluorescent intensities per individual cell. Cut-offs were set for CTCs (CD45-APC mean intensity ≤ 5, DAPI mean intensity >45, CK-PE mean intensity >60, CK-PE size 16-400, CK-PE overlay with DAPI >0.2) and leukocytes (CK-PE ≤ 5, DAPI mean intensity >45, CD45-APC mean intensity >5, CD45-APC size 16-400, CD45-APC overlay with DAPI >0.2). HER-2 scoring is divided in negative (HER-2 mean intensity is 0), medium (HER-2 mean intensity is ≤100), and high (HER-2 mean intensity is >100).

Thirdly, CellSearch®-enriched CTC samples were transferred to the DEPArray™ system (Menarini Silicon Biosystems Inc.), as described previously [18]. Briefly, the loaded sample is automatically injected into the microchamber of a cartridge where single cells are trapped in one of 16,000 electrical cages. IF images of the entire surface area are taken and cells are automatically detected by the system,

generating an image library and 40 parameters per individual. This can be used for further cell sorting.. HER-2-FITC scan filter configuration was set to an exposure time of 800ms with a gain of 5%, empirically defined using cell line samples, and maintained throughout all preclinical and clinical experiments. HER-2 scoring was performed using the relative fluorescent units (RFU) of the HER-2-FITC signal after background subtraction (i.e. Mean Intensity-bgsub parameter). A cut-off for HER-2 positivity was defined at >1185 RFUs. Using this cut-off, 95% of the analyzed cells within the theoretically expected HER-2-positive and -negative cell lines classified as positive and negative, respectively.

HER-2 qRT-PCR on CellSearch® enriched samples

CellSearch® CTC and CellSearch Profile® samples were taken simultaneously to facilitate comparison between IF and gene expression. CellSearch Profile®-enriched tumor cell fractions were subjected to HER-2 expression analysis, as described previously [19]. Tumor cell fractions were lysed in 250µL RNeasy RLT+ buffer (Qiagen BV, The Netherlands) and stored at -80°C until RNA isolation. RNA was isolated from these lysates with the AllPrep DNA/RNA Micro Kit (Qiagen). Complementary DNA (cDNA) synthesis and *ERBB2* pre-amplification was performed on 25% of the isolated RNA, using the RevertAid H Minus First Strand cDNA and TaqMan PreAmp amplification kit, respectively (Thermo Fisher Scientific #K1632 and #4488593, Merelbeke, Belgium), in a GeneAmp® PCR System 9700 (Life technologies). Pre-amplified cDNA was diluted 15x with 1xTE-buffer, after which qRT-PCR was performed to quantitate *ERBB2* transcripts. Additionally, 3 housekeeping genes (*SDHA*, *HMBS* and *HPRT1*) were used to control for sample loading and RNA integrity. Epithelial (*EPCAM, KRT19*) and leukocyte (*PTPRC* coding for CD45) markers were used to control for presence of epithelial and leukocyte content. PCR reactions (40 cycles) were performed using TaqMan Gene Expression Assays with Universal PCR Master Mix No AmpErase UNG on a 7900HT Fast Real-time PCR System (all Applied Biosystems). A calibrator (positive control) sample was used in each run to assess inter-run variability. Negative controls included a NTC and -RT sample. Only samples with a Cq value of <35 for each of the 3 reference genes, were considered of sufficient quality and quantity. *ERBB2* Cq value of every sample

was normalized to the epithelial signal within that sample (dCq). All samples were further normalized to the calibrator (ddCq). RGE was calculated as 2^-ddCq.

HER-2 FISH on CTC

A FISH protocol was established using the preclinical cell line model. CellSearch® enriched cells were spinned on a Superfrost Plus slide (Fisherbrand) using a Slide carrier with a 1ml One-Funnel Cytochamber (cat. 1662 and 1663 resp., Hettich, The Hague, The Netherlands). Cells were fixed on the slides in acetone at 4˚C for 5 minutes. FISH on slides was performed using the DAKO IQFISH kit (Agilent, Diegem, Belgium), including a pretreatment step, denaturation and hybridization, a stringent wash, mounting, and reading. A detailed prescription of the protocol is written in the table below. Before and after FISH, slides were scanned on the BioView® with CTC protocol (BioView®, Israel), in order to detect and map the tumor cells in the leukocyte background before the FISH protocol was applied. Amplification status was scored by the algorithm included in the BioView® software. Furthermore, four patient samples were subjected to HER-2 FISH analysis on BioView®.

Step	Reagent	Concentration	Interval/T	Manufacturer/batch
Slide preparation				
Cytospin			5 min at 800g	Fisherbrand, Superfrost Plus<br>Hettich, #1662 and #1663
Cell fixation	Aceton	1:1	5 min at 4°C	
Add coverslip and scanning	Glycerol	1:1		
Pretreatment				
Remove coverslip				
Cell permeabilization	Dako pretreatment solution 20x	1:20	5 min at 97°C	Agilent, #K5731
Wash	Dako wash buffer 20x	1:20	2x3 min at RT	Agilent, #K5731
Pepsin incubation	Pepsin	1:1	2-3 min at 37°C	Agilent, #K5731
Wash	Dako wash buffer 20x	1:20	2x3 min at RT	Agilent, #K5731
Dehydration	Ethanol	70%, 85%, 100%	3x2 min at RT	Fisher, #BP2818-4
HER-2/CEP17 probe mix	HER2/CEP17 PathVysion probe mix	2:1		Abbott, #06N46-030
Add coverslip and sealant	Fixogum	1:1		Kreatech, #LK-071A
Denaturation and hybridization				
Preheat hybridization chamber			37°C	
Denaturation			5 min at 75°C	
Hybridization			3h at 37°C	
Stringent Wash				
Remove coverslip	Dako stringent wash buffer 20x	1:20		Agilent, #K5731
Wash #1	Dako stringent wash buffer 20x	1:20	10 min at 63°C	Agilent, #K5731
Wash #2	Dako stringent wash buffer 20x	1:20	2x3 min at RT	Agilent, #K5731
Dehydration	Analytical grade ethanol, 100%	70%, 85%, 100%	3x2 min at RT	Fisher, #BP2818-4
Mounting and reading				
Nuclear counterstaining	DAPI	1:1		Abbott, #06N46-030
Add coverslip and sealant	Fixogum	1:1		Kreatech, #LK-071A
